# The Nox1/Nox4 inhibitor attenuates acute lung injury induced by ischemia-reperfusion in mice

**DOI:** 10.1371/journal.pone.0209444

**Published:** 2018-12-20

**Authors:** Yu Cui, Yu Wang, Gen Li, Wan Ma, Xiao-shuang Zhou, Jia Wang, Bin Liu

**Affiliations:** 1 Department of Anesthesiology, West China Hospital of Sichuan University, Chengdu, Sichuan, China; 2 Department of Anesthesiology, Chengdu Women’s and Children’s Central Hospital, Chengdu, Sichuan, China; 3 Department of Anesthesiology, No.363 Hospital, Chengdu, Sichuan, China; 4 Department of Anesthesiology, Vanderbilt University Medical Center, Nashville, Tennessee, United States of America; National Institutes of Health, UNITED STATES

## Abstract

Lung ischemia and reperfusion injury (LIRI) were mediated by several processes including over-production of reactive oxygen species (ROS) and inflammatory activation. ROS generated by nicotinamide adenine dinucletide phosphate (NADPH) oxidase (Nox) may play a pivotal role in pathophysiological changes in a range of disease. However, it was poorly understood in LIRI. Thus, the purpose of our study was to explore whether GKT137831, as a special dual inhibitor of Nox1 and 4, could alleviate LIRI in mice model and explore the minimal dose. According to the protocol, this study was divided into two parts. The first part was to determine the minimal dose of Nox1/4 inhibitor in attenuating LIRI via histopathology and apoptosis analysis. Eighteen C57BL/6J male wild-type mice were randomly divided in to sham, 2.5Nox+sham, 5.0Nox+sham, IR, 2.5Nox+IR and 5.0Nox+IR groups. According to the different group, mice were pretreated with corresponding dose of Nox1/4 inhibitors or normal saline. After LIRI, the results showed 5.0mg/kg Nox1/4 inhibitor could be considered as the minimal dose to alleviate injury by decreasing of lung injury score and the number of TUNEL-positive cells. The second part was to further verify the benefit of 5.0mg/kg Nox1/4 inhibitor in lung protective effects. Thirty-seven C57BL/6J male wild-type mice were divided in to sham, IR and 5.0Nox+IR groups randomly. The results showed that expressions of inflammatory, autophagy cytokines were markedly elevated and PH value was declined after LIRI. However, 5.0 mg/kg Nox1/4 inhibitor significantly attenuated cytokine production as reflected by immunohistochemistry, western blotting and Q-PCR analysis. In conclusion, our findings suggested that 5.0mg/kg Nox1/4 inhibitor contributed to protect lung tissue damage after LIRI via the suppression of inflammatory and autophagy activation.

## 1. Introduction

Lung ischemia reperfusion injury (LIRI), a severe complication which may lead to high morbidity and mortality, is defined as a temporary arrest of ventilation and/or blood supply in at least one lung [1] and paradoxically exacerbates further damage and dysfunction with re-establishment of reperfusion and reoxygenation [2]. LIRI, mainly occurs in lung transplantation or cardiopulmonary bypass, still is one of the greatest challenges in postoperative care, as it can lead to acute lung injury (ALI), even more severe lethal complications such as adult respiratory distress syndrome (ARDS)[3]. A previous animal research demonstrated that no matter short- or long-term changes after LIRI in rats were similar to the changes in ARDS and primary graft dysfunction (PGD) after lung transplantation [4]. Clearly, to date, the current measures are mainly on supportive rather than prophylaxis, and there is no effective method or drug to avoid it’s occurrence in clinical practice.

While LIRI was a complex process, recent research had highlighted the importance in oxidative stress responses, inflammation [1, 5], innate immune responses [2, 6, 7] and endothelial barrier function [2, 7]. When an organ began to suffer hypoxia or ischemia, a series of cascade reactions would be initiated. Although by now no convincing evidence had shown which was the most critical event for tissue damage, increasing evidence suggested that ischemia-reperfusion injury was related to form reactive oxygen species (ROS) [8–10]. Due to the increasing of ROS, the inflammatory process was activated and exacerbated, such as releasing inflammatory cytokines, increasing vascular permeability, platelet aggregation of neutrophils, the occurrence of sterile inflammation, activation of cytokines and the complement system, which had been well summarized previously [11].

NADPH oxidase enzyme (Nox) distributed in tissues and organs widely, which was a membrane protein consisting seven family members (Nox1-5 and Duox1-2), [12]. Increasing evidence had shown that Nox family members took part in a wide range of diseases by producing excess ROS, especially Nox1, Nox2 and Nox4 [13]. Previous reviews had elucidated the structure and function clearly [12, 14, 15]. Recent studies had shown that Nox1 and Nox4 incriminated in inflammation progressing by activating oxidative stress [16, 17].

Therefore, we hypothesize that Nox1/4 inhibitor can alleviate LIRI-associated inflammation, autophagy events and innate immunity. Thus, we conduct the research in mice. We determinate the minimal dose of Nox1/4 inhibitors which can attenuate LIRI. We also test the changes of inflammatory and autophagy cytokines expression, cell apoptosis in *vivo* and measure tissue levels of innate immune response cytokines including interferon-α/β.

## 2. Materials and methods

### 2.1 Nox1/4 inhibitor

GKT137831 was provided by Genkyotex S.A, Geneva, Switzerland, which was a special and efficient dual inhibitor of Nox1/4. It was a pyrazolopyridine dione core inhibitor of the enzymatic activity describing recently, and applied for reducing inflammation in the ischemic retina [18]. In previous research, Nox1/4 inhibitor, GKT137831, was dissolved in normal saline solution, and 60 mg/kg body weight was injected subcutaneously[18]. Thus, in our experiment, GKT137831 was used as Nox1/4 inhibitor.

### 2.2 Animals

Male C57BL/6J wild-type (WT) mice (6–8 weeks old and weighing 20–25 g) were purchased from Sichuan Provincial Laboratory Animal Public Service Center (Chengdu, China). The mice were cured without restriction of water and food in the West China Hospital Laboratory Center. Room temperature and humidity maintained 22 ± 2°C and 55% ± 15% respectively and the light/dark cycle alternated 12:12 hours. This experiment was authorized by the Animal Ethics Committee at West China Hospital of Sichuan University, and the procedure was also supervised under this committee.

### 2.3 Experimental protocol and lung I/R model

The study had been divided into two parts. The first part was to verify whether Nox1/4 inhibitor could alleviate LIRI and determine the minimal dose of Nox1/4 inhibitor by histological and apoptosis analysis. The second part was to find further molecular evidence to determine the minimal dose of Nox1/4 inhibitor could attenuate LIRI.

#### 2.3.1 Grouping

In the first part, C57BL/6J WT mice were allocated to six groups randomly: Group 1 (sham group, n = 3); Group 2 [2.5mg/kg Nox1/4 inhibitors pretreatment group(2.5Nox+sham, n = 3)]; Group 3 [5mg/kg Nox1/4 inhibitors pretreatment group(5.0Nox+sham, n = 3)]; Group 4 (IR group, n = 3); Group 5 [2.5mg/kg Nox1/4 inhibitors pretreatment + IR group (2.5Nox+IR group, n = 3)] and Group 6 (5mg/kg Nox1/4 inhibitors pretreatment group+IR (5.0Nox+IR group, n = 3)].

In the second part, only three group were enrolled, including sham group (n = 12), IR group (n = 12), and 5.0Nox+IR group (n = 13), since we had determined the minimal effective dose.

#### 2.3.2 Experimental protocol

According to different groups, each mouse was pretreated with different dose of Nox1/4 or normal saline, 30min ahead of clamp pulmonary hilum. The Nox1/4 and normal saline were kept in same volume. Anesthetized mice (Intraperitoneal injection of 10% chloral hydrate 0.3 ml/100 g +Fentanyl 0.03mg/kg) were intubated via tracheotomy with 20-guage and mechanically ventilated with FiO_2_ 0.4 by a small animal Minivent (Harvard Apparatus). The setting of respiratory rate was 120 bpm and tidal volume was 200 μL with body temperature at 36.5–37.4°C. Following a left 3-4th lateral intercostal space thoracotomy, 100 U/kg heparin (Qianhong, Changzhou, China) was administrated by intraperitoneal injection. Five minutes later, the left pulmonary hilum, including bronchus, pulmonary artery and vein, was clamped for 60 minutes with a noninvasive micro-vascular clip. Then, the clip was removed in order to re-establish in blood and ventilation for another 120 minutes. The same procedure was performed in the corresponding sham group without clamping pulmonary hilum. After 180 min, arterial blood was drawn from the left ventricle directly. Then mice were euthanized using cervical dislocation under deep anesthesia which was recommended by AVMA [19], and the left lung would be harvested.

### 2.4 Histopathology of lung tissue analysis

Eighteen mice were used to determinate the minimal dose of Nox1/4 inhibitor for alleviating LIRI in six groups (sham, 2.5mg/kg+sham, 5.0mg/kg+sham, IR, 2.5mg/kg+IR, 5.0mg/kg+IR) by histological and apoptosis analysis.

The upper lobes of left lung were fixed in 4% formaldehyde for 24 h, and sent to pathological experiment lab of Sichuan University (Chengdu, China) where the specimen were embedded in paraffin wax, sliced and stained with H&E. Two investigators who were blinded to the grouping completed the histological analysis following the standard of lung injury score [20, 21] under microscope respectively. The final score was determined by the average of two investigators. Briefly, the injury score system was divided into five levels, range from 0(no damage) to 4(severe damage) basing on severity, including alveolar congestion, hemorrhage, interstitial edema, neutrophil count and alveolar wall thickness [20, 21].

### 2.5 Apoptosis analysis

The apoptosis analysis, terminal deoxynucleotidyl transferase dUTP nick end labeling (TUNEL), was conducted according to the manufacturer’s instruction (Millipore, Sigma, Unite States). TUNEL-positive cells with brown color nuclei were counted under the light microscope (×400, Olympus, Japan) by the same investigator who was blinded to the grouping.

### 2.6 Optimal dose of Nox1/4 inhibitor determination

**Fig 1:**
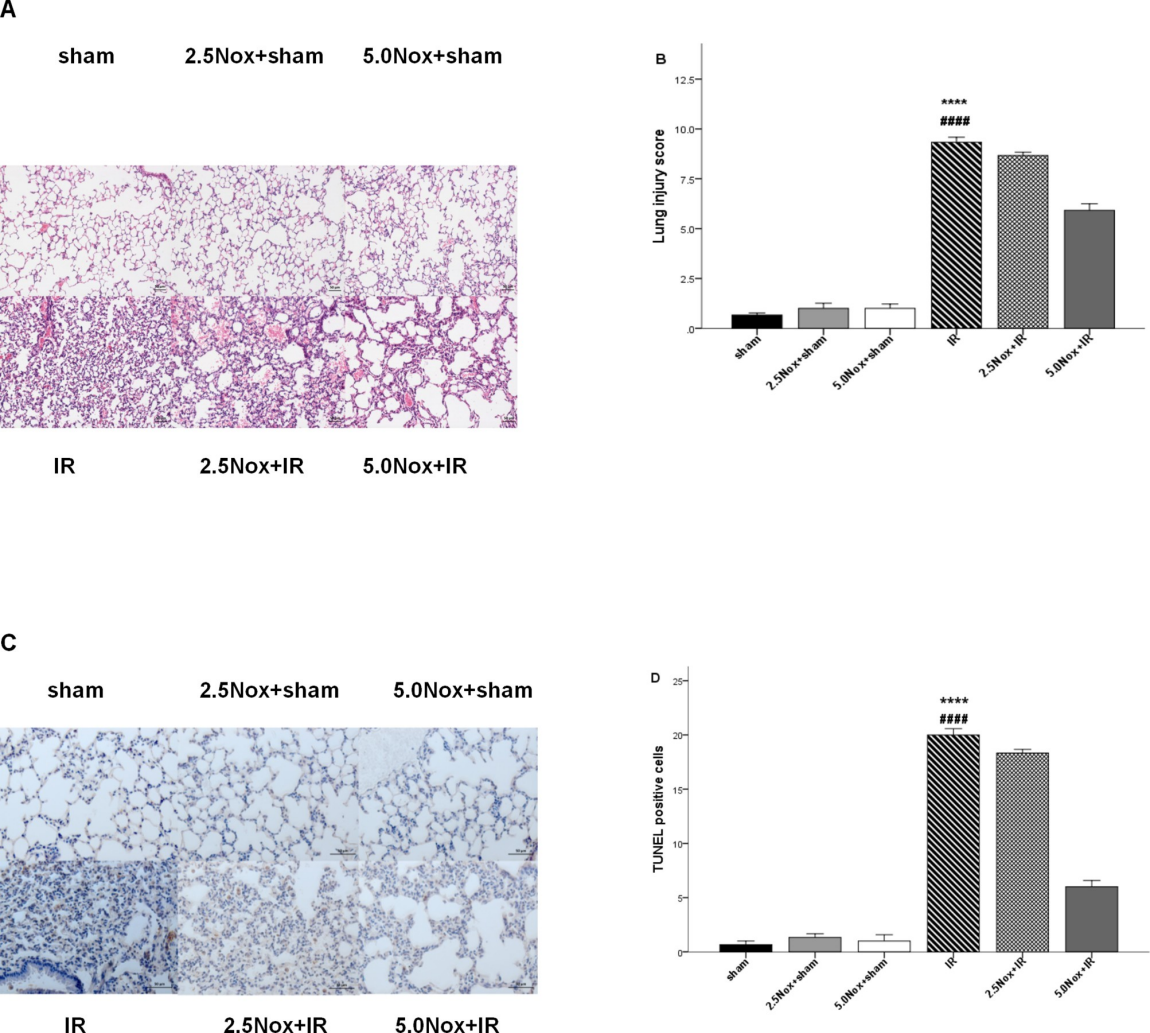
Effects of Nox1/4 inhibitor with different dose pretreatment on LIRI. (A) Representative photomicrographs in HE staining (×400). (B) Histological lung injury was scored as described in the Materials and Methods sections. (C) Lung epithelial cell apoptosis in mice submitted to LIRI. (D) Representative photomicrographs show TUNEL-positive cells (×40). Data were expressed as Mean ± SD (n = 3) and compared by one-way ANOVA; **** *P*< 0.0001 vs. sham, 2.5Nox+sham, 5.0Nox+sham group; ^####^ *P*<0.0001 vs. 5.0Nox+IR group.

Histopathologic and apoptosis evaluation indicated that 5mg/kg Nox1/4 inhibitor pretreatment could be considered the minimal dose in attenuating LIRI (Fig 1A–1D).

**Fig 2:**
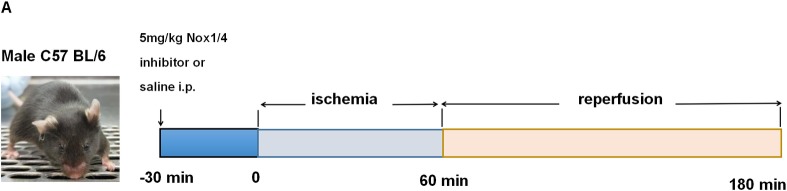
The schematic of LIRI protocol in male C57BL/6J WT mice.

After that, another thirty-seven C57BL/6J WT mice were performed for further analysis. They were randomly divided into three groups (sham = 12, IR = 12, 5.0Nox+IR = 13). The mice were allocated to 5.0 Nox+IR group, and then pretreated with 5.0mg/kg Nox1/4 inhibitor 30 minutes before ischemia. The same procedure was conducted in the sham group with pretreatment of normal saline without clamping pulmonary hilum, and in IR group without any pretreatment (Fig 2).

### 2.7 Arterial blood gas (ABG) analysis

At the end of the procedure, arterial blood was drawn from the left ventricle directly. Then, PH value and PO_2_/FiO_2_ were immediately analyzed by blood gas analyser (Mindray, Shenzhen, China).

### 2.8 Immunohistochemistry analysis

According to the reporting method [22], the nuclear factor-κBp65(NF-κBp65) and TNF-α were analyzed by immunohistochemistry. After embedded, sliced, deparaffinized and washed, the 4–5μm-thick sections were incubated with the rabbit polyclonal anti-NF-κBp65 antibody (catalog no ab16502, 1:500, Abcam, Cambridge,UK) and TNF-α (catalog no ab6671, 1:100, Abcam, Cambridge,UK) respectively. Then, the secondary antibody peroxidase-conjugated goat anti-rabbit IgG (catalog no ZB2301,ZSGB-BIO,Beijing,China) were added at 37°C for 1 hour. Then, the color was displayed with DAB (catalog no ZLI9018, ZSGB-BIO, Beijing, China), and counterstained with hematoxylin. The score system about data expression was calculated according to the reported method with Image J software, Unite Status [23].

### 2.9 Western blotting

Antibodies for α-tublin (catalog no 1122-1-AP,), NF-κBp65 (catalog no 66535-1-LG, Proteintech) and Beclin-1(catalog no ab207612, Abcam) and LC3B(catalog no ab48394, Abcam) were purchased from Unite Status. The procedures were conducted according to manufacturer’s protocol. In brief, the retrieved protein was loaded on SDS-PAGE gel. Next, the protein was transferred to polyvinylidene fluoride membranes (Millipore), and the membranes were incubated with primary antibody (1:1000) against NF-κBp65 at 4°C temperatures overnight. Subsequently, the secondary antibody incubation was conducted with goat anti-rabbit IgG antibody (catalog no 2B2301, ZSGB, China)(1:5000) for 1 h at 4°C temperatures. Finally, the intensity of band was calculated by NIH Image J software, Unite Status.

### 2.10 Quantitative polymerase chain reaction (Q-PCR) analysis

The total RNA extraction was conducted using Trizol Reagent (catalog no 15596026, Ambion, Unite States) in lung tissue. mRNA was reverse-transcripted to cDNA using 5*iScript. RT. Supermix kit (catalog no 64142209, Ambion, Unite States) according to the manufacturer’s instructions. House-keeping gene 18s was used as reference.

In C57BL/6J WT mice, the primer sequences used for qRT-PCR are as follows (from forward to reverse):

IL-6: 5’-CTTCTTGGGACTGATGCTGGT-3’ and 5’-GACTCCAGCTTATCTCTTGGTTG-3′

TNF-α: 5’-CGGGCAGGTCTACTTTGGAG-3’ and 5′-ACCCTGAGCCATAATCCCCT-3′;

Beclin-1: 5’-AGCCTCTGAAACTGGACACG-3’ and 5′-ATGGCTCCTCTCCTGAGTTAG-3′;

LC3B: 5’-GGTTTATGCCTCGCAGGAGA-3’ and 5′-CTGAGTGAAAGGGTGTGGCT-3′

IFNα: 5’-TCAAGCCATCCCTGTCCTACA-3’ and 5′-TCCTGCATCACACAGGCTTTGA-3′

IFNβ: 5’-CCTGGAGCAGCTGAATGGAA-3’ and 5′-GAGTCCGCCTCTGATGCTTA-3′

IFNγ: 5’-TGGAGGAACTGGCAAAAGGA-3’ and 5′-CACTGCAGCTCTGAATGTTTCTTAT-3′

18s: 5’-TTGACTCAACACGGGAAACC-3’ and 5′-AGACAAATCGCTCCACCAAC-3′

### 2.11 Statistical analysis

All results were expressed as Mean ± standard error mean (SEM). Data were analyzed by IBM SPSS 22.0 software. One-way ANOVA and Student *t*-test were used to determine the differences among all groups. *P* values <0.05 was considered as statistically significant.

## 3. Results

### 3.1 5.0mg/kg Nox1/4 inhibitor pretreatment attenuated LIRI

As shown in Fig 1A, there was no obvious difference in histological analysis among sham, 2.5 Nox+sham and 5.0 Nox+sham groups, but clear changes were observed in alveolar congestion, hemorrhage, interstitial edema, neutrophil count, and alveolar wall thickness in corresponding treatment IR groups. However, compared with 2.5mg/kg, 5.0mg/kg Nox1/4 inhibitor pretreatment significantly alleviated the acute damage from LIRI in lung injury score (Fig 1A and 1B). TUNEL staining also proved that LIRI induced to apoptosis of pulmonary tissue in the IR group, but it could be palliated by 5.0mg/kg Nox1/4 inhibitor pretreatment via reducing TUNEL-positive cells. Furthermore, in three sham groups (sham, 2.5Nox+sham, 5.0Nox+sham), the number of TUNEL-positive cells was almost invisible, while a number of TUNEL-positive cells were shown in IR group which could not been alleviated by 2.5mg/kg Nox1/4 inhibitor pretreatment (Fig 1C and 1D). Thus, 5.0mg/kg Nox1/4 inhibitor pretreatment was considered as the minimal dose to attenuate LIRI.

### 3.2 Nox1/4 inhibitor pretreatment improved PH values and PaO_2_/FiO_2_ ratio

**Fig 3:**
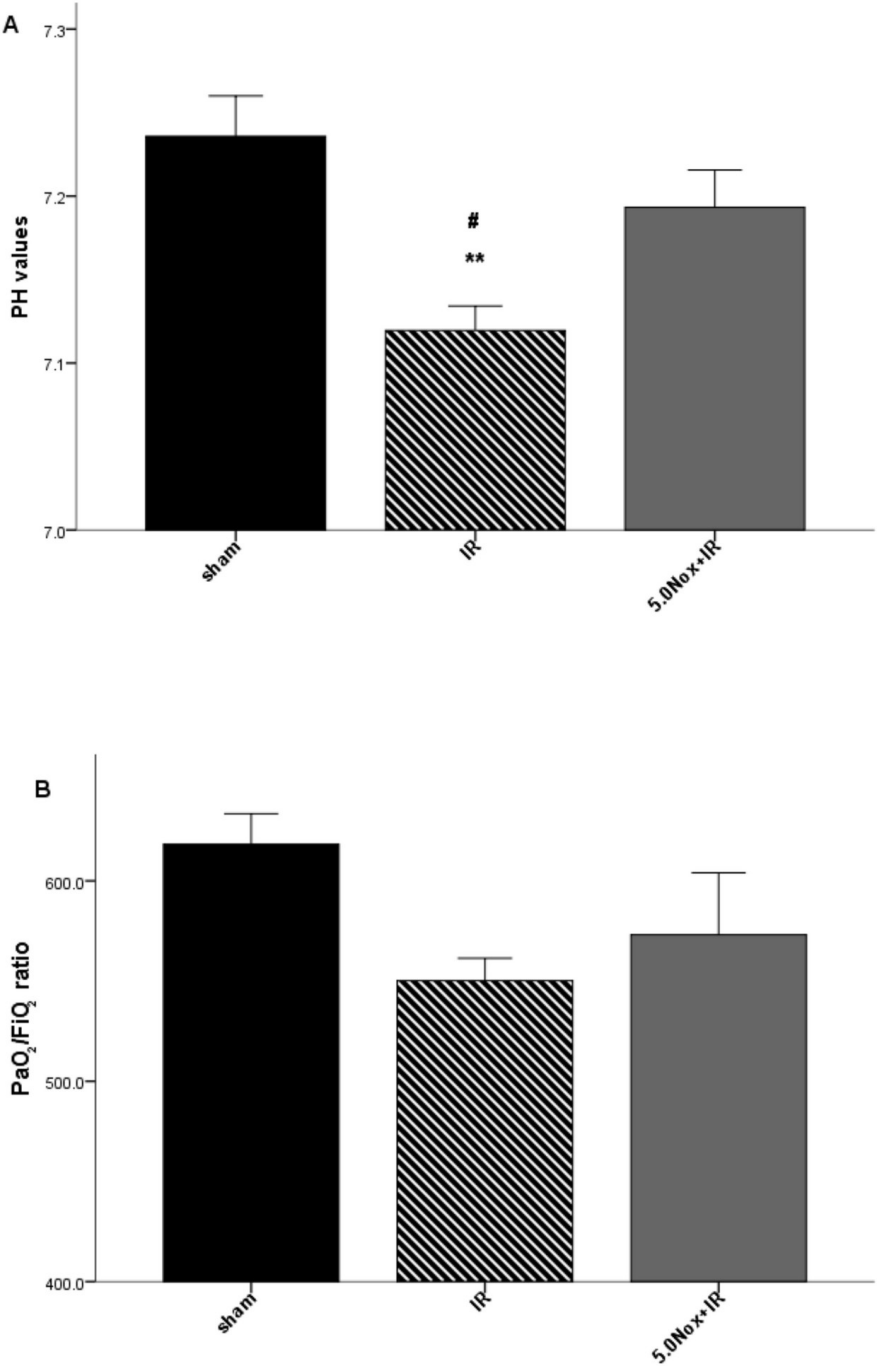
**A, B Effects of 5.0mg/kg Nox1/4 inhibitor pretreatment on PH value and PaO**_**2**_**/FiO**_**2**_ **ratio.** (A) PH values; (B) Arterial blood PaO_2_/FiO_2_ ratios. Data were expressed as Mean ± SEM (n = 6~8) and compared by one-way ANOVA. **P<0.005 vs sham group; ^#^ *P*<0.05 vs 5.0Nox+IR group.

Male C57BL/6J WT mice were pretreated with 5.0mg/kg Nox1/4 inhibitors 30 minutes prior to IR as shown in Fig 2 A. Compared with sham group, there was significant reduction in PH values (7.236 ± 0.241 *vs*. 7.119 ± 0.147, P<0.05) in IR group, but no difference in PaO_2_/FiO_2_ ratio (618 ± 15 *vs*. 550 ± 11, P = 0.053) (Fig 3A and 3B). Reduction of PH values were obviously reversed by 5.0mg/kg Nox1/4 inhibitor pretreatment (Fig 3A), and PaO_2_/FiO_2_ ratio showed an increased trend without statistical difference (Fig 3B).

### 3.3 Nox1/4 inhibitor pretreatment prevented NF-κBp65, TNF α and IL-6 activation after LIRI

**Fig 4:**
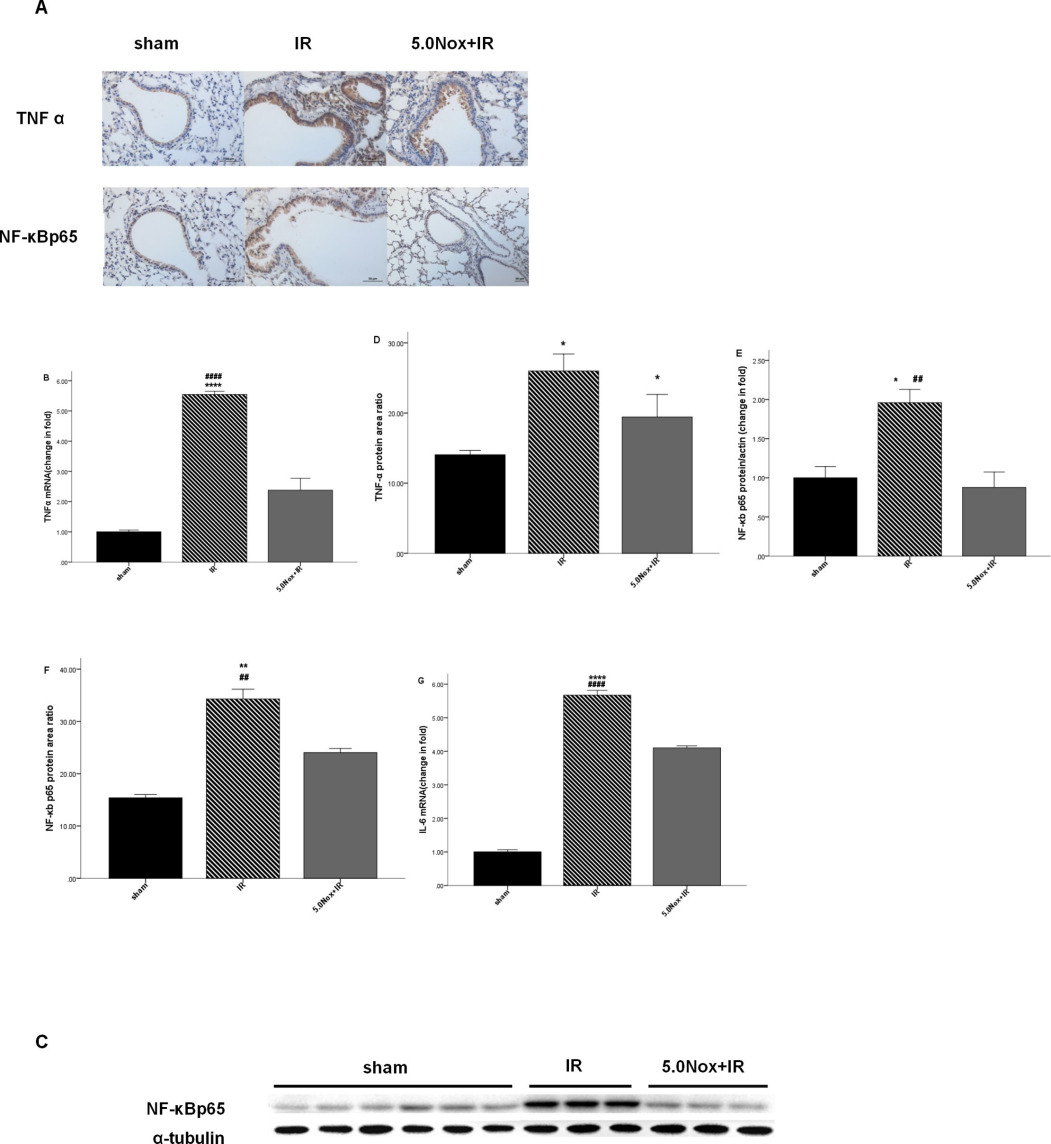
Effects of 5.0mg/kg Nox1/4 inhibitor pretreatment on NF-κBp65, TNF α and IL-6 after LIRI. (A) Immunohistochemical staining (×400) of lung NF-κBp65 and TNF α; (B)The mRNA expression levels of TNF α in lung tissue after LIRI by Q-PCR analysis; (C) Representative Western blotting of NF-κBp65; (D) Calculating area ratio of TNF α in Immunohistochemical staining by Image J software; (E) The protein expression of NF-κBp65 in lung tissue after LIRI by western blotting; (F) Calculating area ratio of NF-κBp65 in Immunohistochemical staining by Image J software; (G) The mRNA expression levels of IL-6 in lung tissue after LIRI by Q-PCR analysis. Data were expressed as Mean ± SEM (n = 6~8) and compared by one-way ANOVA. *P<0.05 vs. sham group; **P<0.005 vs. sham group; ****P<0.0001 vs. sham group; #.

The two kinds of technologies were applied to determine that NF-κBp65 and TNF-α activation were prevented after 5.0mg/kg Nox1/4 inhibitor pretreatment in LIRI (Fig 4A–4F). As shown in Fig 4A, 4D and 4F, NF-κBp65 and TNF-α immunohistochemistry staining expression were higher in IR and 5.0Nox+IR group compared with the sham group. 5.0mg/kg Nox1/4 inhibitor pretreatment significantly decreased the level NF-κBp65 in lung tissue, which were confirmed by western blotting test (Fig 4C and 4E). In contrast, 5.0mg/kg Nox1/4 inhibitor pretreatment did not significantly reduce TNF-α expression in immunohistochemical assays, except for a downward trend. However, this difference was confirmed by the subsequent Q-PCR analysis which was more accurate (Fig 4B and 4D). Moreover, IL-6 mRNA concentration in IR group was higher than those of sham and 5.0Nox+IR groups (Fig 4G).

### 3.4 Nox1/4 inhibitor pretreatment prevented LIRI-induced autophagy activity in lung tissue

**Fig 5:**
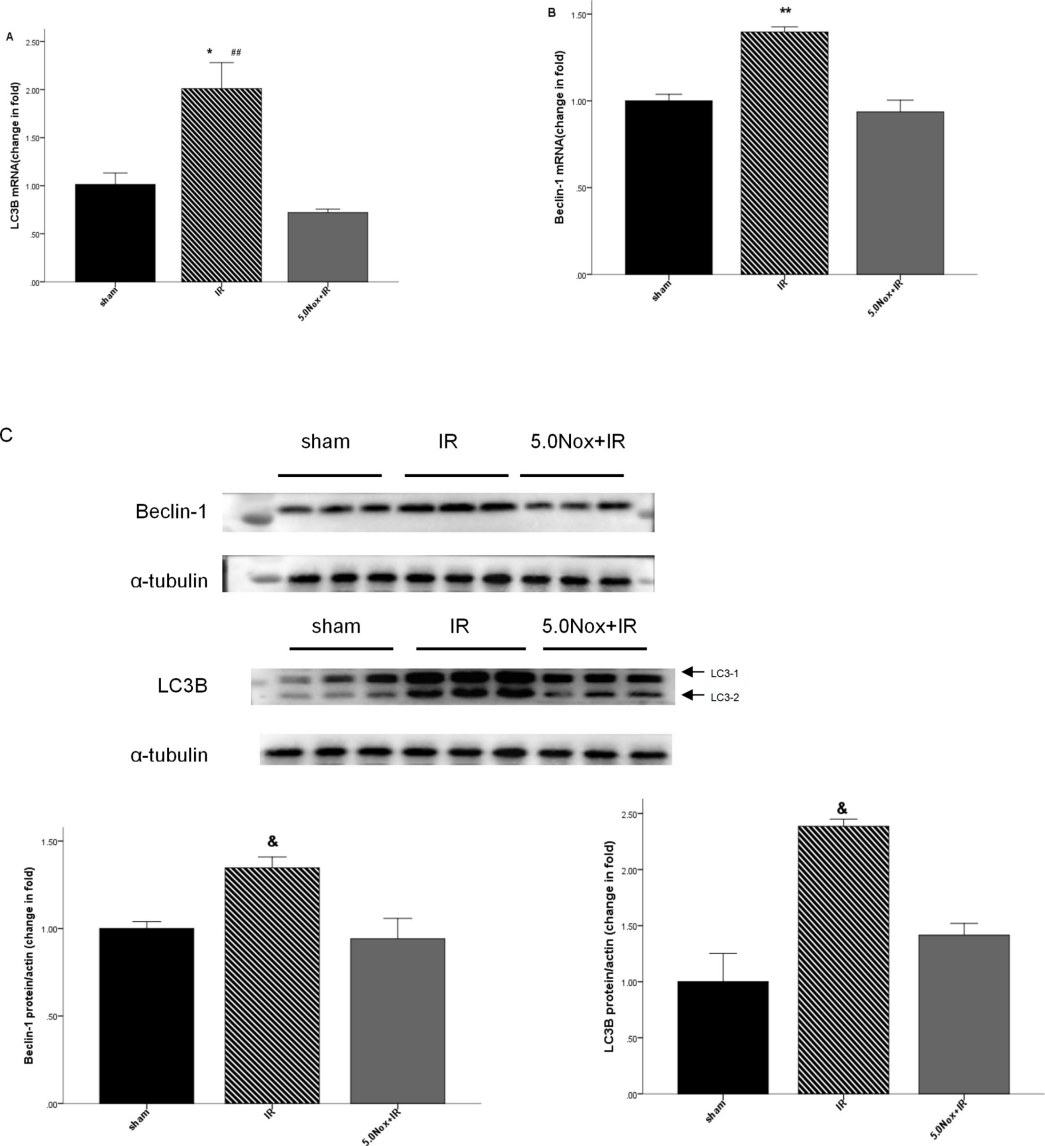
Effects of 5.0mg/kg Nox1/4 inhibitor pretreatment on LC3B and Beclin-1 mRNA expression after LIRI. (A)The mRNA expression levels of LC3B in lung tissue after LIRI by Q-PCR analysis; (B) The mRNA expression levels of Beclin-1 in lung tissue after LIRI by Q-PCR analysis; (C) The protein expression of Beclin-1 and LC3B in lung tissue after LIRI by western blotting. Data were expressed as Mean ± SEM (n = 6~8) and compared by one-way ANOVA. *P<0.05 vs. sham group; ^&^ *P*<0.05 vs. sham group or 5.0Nox+IR group; **P<0.005 vs. sham group or 5.0Nox+IR group; ^##^*P*<0.005 vs. 5.0Nox+IR group.

To demonstrate whether Nox1/4 Inhibitor pretreatment associated with autophagy induced by LIRI, the expression level of LC3B and Beclin-1 were tested in this study. The results determined that both LC3B and Beclin-1 were significantly increased after LIRI in lung tissue, while they could be reduced by 5.0mg/kg Nox1/4 Inhibitor pretreatment (Fig 5A, 5B and 5C). The results indicated that 5.0mg/kg Nox1/4 Inhibitor may confer lung protective effect via inhibiting autophagy activation.

### 3.5 Effects of Nox1/4 inhibitor disposal on immune related cytokines IFN α, IFN β and IFNγ

**Fig 6:**
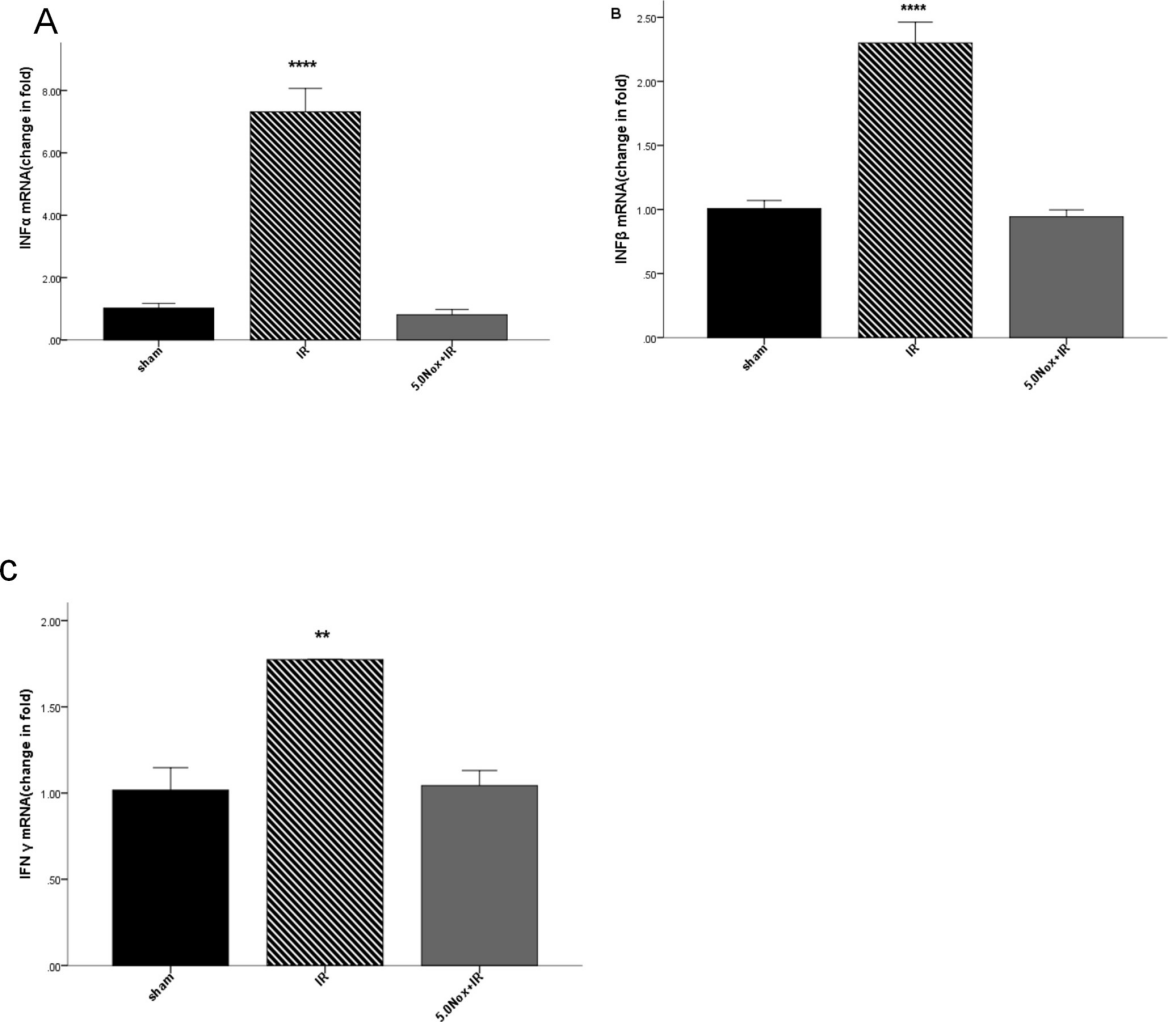
Effects of 5.0mg/kg Nox1/4 inhibitor pretreatment on IFN α/β/γ mRNA expression after LIRI. (A) The mRNA expression levels of IFN α by Q-PCR analysis; (B) The mRNA expression levels of IFN β Q-PCR analysis; (C) The mRNA expression levels of IFNγ by Q-PCR analysis. Data were expressed as Mean ± SEM (n = 6~8) and compared by one-way ANOVA. ***P<0.0005 vs. sham group or 5.0Nox+IR group; ****P<0.0001 vs. sham group or 5.0Nox+IR group.

A previous study had proved IFN α and IFN β expression increased in inflammatory setting induced by hypoxia [24]. The expression of IFNγ was decreased when lung warm ischemia reperfusion injury was attenuated [25]. Q-PCR analysis demonstrated a significant upregulation IFN α, IFN β and IFNγ (7.32 ± 0.75, 2.30 ± 0.16 and 1.77 ± 0.00 fold respectively) after 60 minutes ischemia and 120 minutes reperfusion (Fig 6A, 6B and 6C), and 5.0mg/kg Nox1/4 inhibitor pretreatment dramatically decreased the levels of them. The above results confirmed that the lung protective effects of Nox1/4 inhibitor may be regulated via the regulation of immune function.

## 4. Discussion

In the current study, we tested whether different dose of Nox1/4 inhibitors had a different function in the progress of lung ischemia reperfusion injury in mice model. Our finding demonstrated that 5.0mg/kg Nox1/4 inhibitors pretreatment could mitigate lung injury after ischemia and reperfusion, while 2.5mg/kg had no significant lung protective effects. Histopathological and apoptosis results showed a significant lung injury after ischemia and reperfusion, but they were palliated by pretreatment of 5.0mg/kg Nox1/4 inhibitors. The benefit of 5.0mg/kg Nox1/4 inhibitor was further verified by improving acidosis and downregulating of inflammation and autophagy cytokines expression. In addition, IFN α and IFN β and IFNγ, involving the regulation of pro-inflammatory cytokines [24], were significantly upregulated after LIRI, but those trends were also controlled by 5.0mg/kg Nox1/4 inhibitor disposal. The present study showed a potential interrelationship between Nox1/4 inhibitor pretreatment and the progress of LIRI.

It was well known that LIRI involved a series of pathological reactions including oxidative stress, inflammation, autophagy and innate immunity [2, 26, 27]. Increasing evidence showed ROS generated by Nox could lead to inflammatory damage through the expression of transcription activator and inflammatory factors, including NF-κB p65, IL-6 and TNFα. Nox4 KO reduced infract size after cardiac ischemia-reperfusion and Nox1/4 inhibitor administration played anti-inflammation via reducing ROS level in ischemia retina [28, 29]. Over-production of ROS had been proven to involve LIRI and inflammatory processes [30]. The severity of lung injury was assessed by alveolar congestion, hemorrhage, interstitial edema, neutrophil count and alveolar wall thickness [20, 21]. Indeed, that Nox1/4 inhibitor was a kind of substance to avoid ROS generation was well-known, but whether the benefit of it could extend to protect LIRI and the minimal dose were poorly understood. According to existing literature, the dose of Nox1/4 inhibitor to inhibit NADPH-dependent ROS production could be ranging from 5–60 mg/kg p.o [31]. However, because of the higher bioavailability by i.p, we started the dose from 2.5 mg/kg in order to obtain the minimal dose for mitigating LIRI. Consisted with previous research, our data also confirmed that 5.0 mg/kg Nox1/4 inhibitor administration apparently reduced lung injury score and cell apoptosis. Thus, we proposed that 5.0mg/kg Nox1/4 inhibitor was a minimal dose in attenuating LIRI. However, the above results were not enough to prove the lung protective effects by Nox1/4 inhibitor. Next, we further explored the lung protective effects of 5.0mg/kg Nox1/4 inhibitor through molecular mechanisms. Fortunately, we also determined the level of NF-κB, TNF α, IL-6 was dramatically upregulated after LIRI and downregulated after 5.0mg/kg pretreatment respectively. All of above results proved 5.0 mg/kg Nox1/4 inhibitor pretreatment had the effects of anti-inflammation in LIRI model.

Hypercapnic was not easily avoided after LIRI or long-time mechanical ventilation. Kavanagh’s group first demonstrated the benefit effects of hypercapnic after LIRI in the isolated rat lung [32]. However, there was no consensus on an acceptable lower limit for pH value. Compared with other groups, our study showed the lower PH value in IR group with more severe lung injury. The contradiction may due to limited PH value with protective effect, and beyond it may have opposite effects. Thus, we speculated moderate hypercapnic had protective effects after LIRI, but more severe hypercapnic would reduce or eliminate the benefits, which was similar with previous study [33]. Therefore, further experiments should be conducted to obtain the lowest protective PH value. Moreover, it was well known the serious LIRI was always accompanied pulmonary dysfunction. In clinical practice, PaO_2_/FiO_2_ ratio was an indicator to reflect the severity of lung injury. Interestingly, PaO_2_/FiO_2_ did not show any statistical difference between three groups in this study, only with a decreasing trend in IR group. We attributed it to the pulmonary function was still within compensative scope, even after ischemia-reperfusion, which was similar in clinical practice [34].

Moreover, we also observed the expression of IFN α and IFN β obviously increased after 3h ischemia-reperfusion, compared with Nox1/4 inhibitor pretreatment group in this experiment. As mentioned in existed research, type-1 IFNs, including IFN α and IFN β, could induce pro-inflammation resulting in secretion of inflammatory cytokines. Minter and his colleagues found increasing type -1 IFN deteriorated the hypoxic-ischemic injury outcomes in neuro cells [24], which implied IFN α and IFN β may involve in inflammatory activation. We proposed ROS may also associate with it, because Nox1/4 inhibitor could decrease the level of IFN α and IFN β dramatically. Furthermore, Martens et al. reported that the level of IFNγwas increased after lung warm ischemia reperfusion injury [25]. In our study, the similar trend was observed. However, little was known about the potential mechanism. More research was needed to illuminate the relationship among IFN α, IFN β, IFNγ and secretion of inflammatory cytokines.

Although a great deal of work had been devoted to investigate the role of autophagy in LIRI, it remained many controversies about the exact role of autophagy. Autophagy, as a normal physiological process, was regarded as involving degradation of cytoplastic protein and molecules by autolysosome. Emerging evidence had proved dysfunction of autophagy was associated with ameliorating LIRI [35]. Correspondingly, over-expression of autophagy had a relationship with enhancing lung cell apoptosis [36], which was demonstrated in CPR and LIRI processes [37]. The expression of LC3B and Beclin-1, both as definitive autophagy biomarkers, were increased after lung transplantation which also was involved in LIRI in rats [36]. The level of LC3B and Beclin-1 increased at 3 hours after lung transplantation, achieved peak point at 6 hours and then decreased [38]. In our study, we also found the excessive protein and mRNA expression of LC3B and Beclin-1 after 3h LIRI and then dramatically inhibited by pretreatment 5.0mg/kg Nox1/4 inhibitor, which signified excessive autophagy may be restricted by pretreatment 5.0mg/kg Nox1/4 inhibitor in mice LIRI model.

Furthermore, although chloral hydrate for anesthesia or euthanasia was un-recommended by international laboratory animal science/veterinary entities as it was regarded to produce sedation rather than anesthesia and caused marked respiratory depression [39], we chose Intraperitoneal injection of 10% chloral hydrate 0.3 ml/100 g combined with 0.03mg/kg fentanyl, which offered good analgesic potency and sedation for mice. Opioids were usually combined with tranquilizers so that the amount of tranquilizer needed for heavy sedation could be reduced, decreasing unwanted side effects [40]. Another important reason was that 10% chloral hydrate had been used in our previous research. We still chose it for sedation in order to minimize multiple exposures and balance with previous studies, but we combined with fentanyl for analgesia for minimizing mouse suffering. Moreover, in our study procedure, the anesthesized mice received mechanical ventilation immediately, so the respiratory depression was not a big issue. In the future, we will pay much more attention to the euthanasia issues and scientific animal welfare methods.

There also had some limitations in our study. We did not test the change of expression of Nox1 and Nox4 after LIRI. Although G137831 was a kind of dual inhibitor of Nox1 and Nox4 [29], the degree of inhibition was unknown in mice LIRI model. In addition, the detailed mechanisms should be investigated to clarify how Nox1/4 inhibitor played the protective effects in LIRI. In further research, we will focus on the underlying mechanisms not only on decreasing inflammatory and autophagy cytokines, but also the potential pathway of oxidative stress and innate immunity.

## 5. Conclusions

In conclusion, 5.0mg/kg Nox1/4 inhibitor can alleviate LIRI by downregulating the expression of inflammatory and autophagy cytokines. The apoptosis also has been inhibited after administration of Nox1/4 inhibitor. The innate immune and oxidative stress may also involve in the lung-protective mechanisms, but further research should be conduct to explore the potential pathways on how Nox1/4 inhibitor contributes to lung-protective effects.

## Abbreviations

LIRI: Lung ischemia and reperfusion injury
ROS: Reactive oxygen species
NADPH: Nicotinamide adenine dinucletide phosphate
Nox: Nicotinamide adenine dinucletide phosphate oxidase
ALI: Acute lung injury
ARDS: Adult respiratory distress syndrome
PGD: Primary graft dysfunction
WT: Wild type
TUNEL: Terminal deoxynucleotidyl transferase dUTP nick end labeling
ABG: Arterial blood gas
NF: κBp65, Nuclear factor-κBp65
PCR: Quantitative polymerase chain reaction
TNF-α: Tumor necrosis factor α
IL-6: Interleukin 6
